# Patient derived cancer-associated fibroblasts from non-small cell lung cancer undergo phenotypic drift in culture

**DOI:** 10.1038/s44276-025-00159-w

**Published:** 2025-07-10

**Authors:** Layla Mathieson, Phoebe Jones, Lilian Koppensteiner, Liam Neilson, David A. Dorward, Richard O’Connor, Ahsan R. Akram

**Affiliations:** 1 Centre for Inflammation Research, Institute of Regeneration and Repair, University of Edinburgh, Edinburgh, UK; 2 Department of Pathology, Royal Infirmary of Edinburgh, Edinburgh, UK; 3 Cancer Research UK Scotland Centre, Institute of Genetics & Cancer, The University of Edinburgh, Edinburgh, UK

## Abstract

**Background:**

Cancer-associated fibroblasts (CAFs) are the predominant cell type in the stroma of many solid organ malignancies, including non-small cell lung cancer (NSCLC). They exhibit considerable phenotypic and functional heterogeneity and are widely used in functional assays and co-culture models. CAF research frequently involves the in vitro expansion and maintenance of CAFs to facilitate functional assays and co-culture studies. However, less is known about how in vitro culture temporally affects CAF phenotype.

**Methods:**

We characterised the phenotype of CAFs from NSCLC patients compared to non-cancerous lung fibroblasts using conventional in vitro conditions by tracking changes in CAF subset marker expression levels by flow cytometry. Additional transcriptomic and functional analyses were performed to determine differences between CAFs and non-cancerous fibroblasts.

**Results:**

We demonstrate that CAFs from NSCLC undergo phenotypic drift in culture, and that there is a convergence to a subset phenotype predominantly upregulated in non-cancerous lung. Additionally, we demonstrate the phenotype, transcriptome and function of fibroblasts converge between CAFs and fibroblasts from non-cancerous lung by the third culture passage, suggesting that in vitro conditions promote this phenotype.

**Conclusion:**

We highlight the need to understand and monitor the culture phenotype during functional studies with CAFs, as the heterogeneity found in the tumour microenvironment is rapidly lost in cultured cells.

## Introduction

Cancer associated fibroblasts (CAFs) are a crucial element of the tumour microenvironment in solid organ cancers [1]. In contrast to fibroblasts activated during physiological processes such as wound repair, CAFs have increased proliferative ability as well as an upregulated secretory profile [2]. CAFs contribute to multiple aspects of tumour development, including metastasis, invasion, angiogenesis, immune evasion and resistance to therapies [3–5]. The ability to study CAFs ex vivo is therefore of critical importance in cancer research, and allows the study of CAF functionality as well as supporting development of imaging modalities to target CAFs.

CAFs are not a uniform population; instead, they encompass diverse subtypes with distinct molecular, spatial, and functional characteristics. We have previously identified five CAF subtypes in non-small cell lung cancer (NSCLC) based on flow cytometry markers, each with prognostic significance [6]. These findings are supported by studies utilising single-cell RNA sequencing, spatial transcriptomics, and imaging mass cytometry, which have revealed functionally and prognostically distinct CAF states, often classified as myofibroblastic, inflammatory, and antigen-presenting CAFs [7–10]. These insights have catalysed new frameworks for understanding CAF biology and for developing therapeutic strategies targeting specific CAF subsets.

Multiple methods of culturing CAFs have been employed in other studies, including 2D culture, growth from tissue slices, spheroids and organoids [11–14]. The most common method is standard 2D culture, where fibroblasts are grown from single cell suspensions of digested tumour tissue in tissue culture flasks [15]. Cells readily adhere to tissue culture plastic can be expanded efficiently and cost-effectively, for further study. There are, however, limitations with this culture method, as CAFs are grown in isolation from other cell types resulting in loss of niche pressures. The phenomenon of phenotypic drift is widely accepted in tissue culture, with various studies showing that different cell types, both cell lines and primary cells, change phenotype in culture [16–20]. It has also been acknowledged previously that CAFs require detailed characterisation and investigation into their ability to maintain functions in culture [21]. Despite this, a large proportion of studies rely on cultured cells remain a predominant source for the study of cell function and in drug discovery. Given the advances in CAF research and discovery that CAFs represent a heterogeneous population in vivo in many solid organ cancers including breast [22, 23], ovarian [24], pancreatic [7] and lung cancers [6, 25], it is important to understand the changes in culture, and the relation of a culture phenotype to that of the in vivo phenotype.

In this study, we sought to understand the changes in phenotype of CAFs and non-cancerous lung fibroblasts (NF) derived from human non-small cell lung cancer (NSCLC) throughout culture. We assess the changing levels of cell surface markers, functional changes, and transcriptional changes from initial isolation to cultured cells and compare this across cells derived from tumours and adjacent non-cancerous lung from patients with non-small cell lung cancer.

## Methods

### Ethics statement

Cancerous and adjacent non-malignant lung tissues were obtained with ethical approval from the NHS Lothian Research Ethics Committee (REC No: 15/ES/0094), facilitated by NHS Lothian SAHSC Bioresource. Written informed consent was obtained from all individuals prior to tissue collection.

### Digestion of NSCLC samples

Tumour tissue and adjacent non-cancerous lung tissues were obtained from patients with NSCLC undergoing surgery with curative intent. Tumour areas were identified and sampled by a thoracic pathologist and samples also taken from the most distal region of the resection for non-cancerous lung. Tissues were processed and digested to single cell suspension as previously described [6]. Briefly, samples were mechanically dissociated with forceps and enzymatically digested with collagenase I [1 mg/ml] (Gibco) and DNase [0.1 mg/ml] (Sigma) for 1 h followed by 10 min further digestion with TryplE express (Gibco). Resulting suspensions were passed through a 70 μm cell strainer to isolate single cells, and erythrocytes lysed using red blood cell lysis buffer (Roche).

### Culture of primary fibroblasts

Single cell suspensions following tissue digest were transferred to T75 flasks with 15 ml prewarmed complete media. The medium composition was based previous studies to culture human and murine CAFs [6, 26–33]: DMEM (Gibco) containing 100 U/L penicillin-streptomycin (Gibco), 2mM L-glutamine (Gibco), 10% foetal bovine serum (Life Science Production) and 1x Insulin-Transferrin-Selenium (Gibco). After 24 h media was changed to remove debris and non-adherent cells. Cells were incubated at 37 °C, 5% CO_2_ and media changed twice weekly until confluence, when cells were passaged. After washing cells twice with pre-warmed PBS, trypsin was used to detach cells from flasks. After addition of complete media to halt trypsin action, the resulting suspension was centrifuged at 300 × *g* for 5 min and the cells could then be counted for re-seeding or experimentation.

### Flow cytometry

Cells were stained as previously described [6]. Briefly, 1 million cells per condition were stained with the live/dead viability dye Zombie UV (1:1000, Biolegend) for 30 min at room temperature in the dark. Cells were then washed (FACs buffer, DPBS (Gibco) supplemented with 2% FBS), and blocked using FC blocker (Biolegend) before being stained with surface antibodies (EpCAM, CD45, CD31, FAP, CD29, Podoplanin and PDGFRβ) or corresponding isotype controls (see Supplementary Table S1) for 20 min at 4 °C. Cells were fixed using Cytofix fixation buffer (BD Biosciences) for 20 min and permeabilised in Perm/Wash buffer (BD Biosciences). Intracellular antibodies (αSMA and FSP-1) or the corresponding isotype controls were added to cells and incubated for 30 min at 4 °C. After washing, cells were stored in FACs buffer overnight at 4 °C before data acquisition on a LSR6Fortessa analyser (BD Biosciences). Compensation was performed using single stain control UltraComp eBeads (Invitrogen).

### Flow cytometry data analysis

Flow data was analysed using FlowJo version 10.7.1. Fibroblasts were defined as cells which were live, EpCAM-, CD45- and CD31- to exclude epithelial, leucocytes and endothelial cells respectively (Supplementary Fig. S1). Subsets were determined by applying the FlowSOM analysis described previously [21] to determine distribution of CAFs across five CAF subsets (termed CAF S1–S5).

### Cell sorting

Cells were stained with lineage exclusion markers (EpCAM, CD31, CD45; all conjugated to BV605) as described above, and maintained on ice prior to sorting. DAPI was added immediately before sorting. Viable, lineage-negative cells were sorted using a BD FACSAria Fusion cell sorter directly into RNA-free microcentrifuge tubes for downstream processing.

### RNA processing

Sorted fibroblasts were centrifuged at 400 × *g* for 10 min and supernatant removed using RNA free pipette tips. The cell pellets were then resuspended in RLT buffer (Qiagen), at a concentration of 5000 cells/5 μl, incubated incubated on ice for 20 min to allow cell lysis, and stored at −80 °C. Samples were University of Edinburgh HTPU Facility for analysis using the NanoString Human PanCancer Progression panel to assess genes associated with extracellular matrix remodelling (ECM) and epithelial-to-mesenchymal transition. Reporter codesets were prepared by adding 70 μl of hybridisation buffer and 2.1 μl proteinase K. Tubes were added to a PCR block pre-heated to 65 °C and incubated for 18 h. Hybridised samples and all components of the nCounter masterkit were then processed using the high sensitivity protocol. Samples were then assigned to the sample cartridge and read in the digital analyser set to 555 fields of view.

### RNA expression analysis

Analysis files were uploaded to Rosalind (https://rosalind.bio/) for analysis by the Nanostring Advanced Analysis protocol. QC was performed and four samples were excluded from analysis due to a binding density flag. The remaining samples were normalised using a selection of housekeeping genes, selected for normalisation were those with the least variance, as determined by the geNorm algorithm implemented in the NormqPCR R library [34]. Differential expression was calculated for the groups of interest. Fold changes and P values were calculated using the fast method as recommended by nanostring, and p-value adjustment was calculated using the Benjamini-Hochberg method to estimate false discovery rates. For KEGG analysis normalised counts data from bulk RNA -seq was exported to R Studio (v.2024.12.1 + 563, Posit Software, PBC). The R tidyverse package (v.2.0.0) was then used to prepare sample metadata for analysis. The R limma package (3.62.2) was then used to perform differential gene analysis between normal digest and normal P3 samples and tumour digest and tumour P3 samples. Data was then prepared for KEGG analysis using the clusterProfiler package (v.4.14.6) to assign gene names using human specific genome wide annotation package, org.Hs.eg.db package (v.3.20.0). KEGG analysis for key stress pathways (HIF-1, PI3K, ECM, cellular senescence) and autophagy were then performed using the clusterProfiler, org.Hs.eg.db, and dplyr (v.1.1.4) packages. KEGG analysis of all pathways was visualised as dotplots for both normal and tumour separately using ggplot2 (v3.5.2). Additionally, a network plot was produced to determine what genes from each pathway were differentially express using clusterProfiler and org.Hs.eg.db packages.

### Transwell migration assay

To assess migratory ability in response to a chemotaxis gradient, fibroblasts were collected in suspension at a density of 2.5 × 10^4^ cells/ml in complete media and 0.5 ml of suspension added to the apical chamber of the transwell insert (Corning Falcon cell culture inserts, 12 well, 8 μm pore size). 1 ml of complete media (10% FBS) was added to the basal chamber. After 24 h to allow cell adhesion to occur, all media were removed and 1 ml of complete media added to the basal chamber and 0.5 ml serum free media was added to the apical chamber.

After 48 h, cells were fixed and stained with 0.5% crystal violet for 10 min. Non-migrated cells were removed using DPBS-soaked swabs. Stained cells were visualised on an EVOS brightfield microscope. To quantify migration, crystal violet was eluted with 33% acetic acid and absorbance measured at 590 nm using a BioTek Synergy plate reader.

### Collagen contraction assay

To assess the contractile ability of CAFs and NFs, 3 × 10^4^ CAFs were embedded in 100 µl of 2.5 µg/ml collagen solution (*Corning, Collagen Type I, Rat Tail High Concentration, 10.57* *mg/ml*) in a 96-well flat bottom plate. After 30 min of incubation at 37 °C to allow polymerization, 100 µl complete media was added to each well. Each condition was repeated in triplicate. 100 µl PBS was added to the surrounding wells to reduce evaporation.

Contraction was assessed after 48 h. The wells were imaged using a dissection microscope and the percentage of contraction was quantified on ImageJ software. The formula ($\frac{{area\; of\; well}-{area\; of\; contracted\; gel}}{{area\; of\; well}}X100$) was used to calculate the percentage contraction. Statistical analysis was carried out on GraphPad Prism version 10.1.0.

### Wound healing assay

To assess fibroblast migratory ability, CAFs and NFs were collected in suspension and seeded in silicon culture inserts with a 500 µm chamber separation (Ibidi Catalogue #80209) at a density of 5 × 10^4^ cells/ml (70 µl per chamber). After 24 h, inserts were removed and media changed in the well following washing cells with pre-warmed DPBS. The cell free gap was imaged using an EVOS brightfield microscope at 0, 24 and 48 h. Images were analysed in ImageJ, with the plugin wound_healing_size_tool.ijm [35] utilised to calculate the cell free area of each image.

### Secretome Assessment

Conditioned media was collected from cultures 48 h after media replacement at ~70% confluence were centrifuged at 350 × *g* for 10 min to remove cells. A set of 13 analytes (TGF-β1, IL-6, GM-CSF, IL-33, CCL2, CCL3, CCL4, CCL20, CXCL8, CXCL9, CXCL10, CXCL11, CXCL12) was quantified according to manufacturers’ instructions, using a custom Legendplex-assay (LEGENDplex^TM^ Biolegend). Data collection was performed according to manufacturer’s instructions using the Attune NxT Autosampler and analysis was performed using LEGENDplex^TM^ data analysis software. LIF ELISAs (R&D) were performed according to manufacturers’ instructions.

### Statistical analysis

Patient-derived samples were collected in an unbiased manner and in accordance with approved ethical protocols. All statistical analyses were performed using GraphPad Prism (version 10). Data are presented as mean ± standard deviation (SD), unless otherwise stated. Where statistical comparisons were performed, one-way ANOVA followed by Tukey’s multiple comparisons test or uncorrected Fisher’s LSD test was used, as appropriate. Statistical significance was defined as *p* < 0.05, with levels of significance indicated as follows: *p < 0.05, **p < 0.01, ***p < 0.005, ****p < 0.001.

## Results

**Fig. 1:**
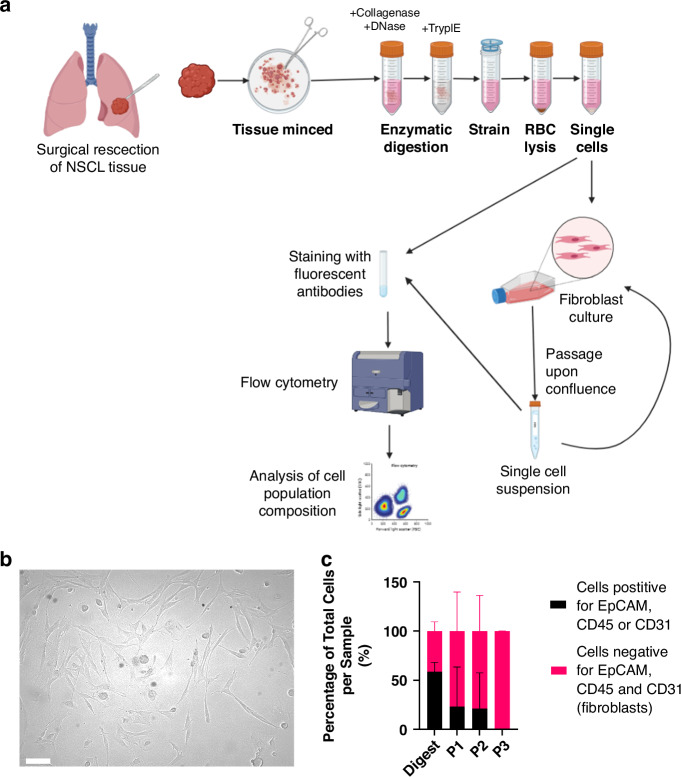
Fibroblasts predominate in primary human non-small cell lung cancer culture from tissue resections by passage 3. **a** Protocol for digestion and culture of fibroblasts, with analysis by flow cytometry at different stages of culture; **b** Representative image of fibroblasts growing in culture, scale bar represents 50 μm; **c** Proportion of cells in tumour samples which are positive for lineage markers EpCAM, CD45 or CD31 compared to proportion which are fibroblasts demonstrating by passage 3 fibroblasts dominate in culture. Error bars show standard deviation. *N* = 3.

Fibroblasts were successfully cultured from paired surgically resected NSCLC tissue samples and tumour-adjacent non-cancerous lung (NCL) tissues to enable matched comparison of fibroblasts from both origins (Fig. 1a). As predominate in vitro in culture, single cell suspensions were seeded in culture flasks and as media was changed to remove non-adherent cells, fibroblast cultures were established (representative image of fibroblasts in culture Fig. 1b). To assess the proportion of fibroblasts within the culture, cells were stained with lineage markers EpCAM, CD45 and CD31 to identify epithelial cells, leucocytes, and endothelial cells respectively, and lineage negative cells were considered fibroblasts. This revealed that fibroblasts quickly constitute the majority of the population in culture, and by passage 3 they remain the only cell type in the culture (Fig. 1c).

Fibroblasts represent a highly heterogeneous population, demonstrated by variability in the expression level of activation markers analysed through flow cytometry. Prior work from our group has classified these fibroblasts into five distinct subsets (CAF S1–S5) based on differential expression of key markers, including fibroblast activation protein (FAP), podoplanin (PDPN), α-smooth muscle actin (αSMA), fibroblast-specific protein 1 (FSP-1), platelet-derived growth factor receptor beta (PDGFRβ), and CD29. This prior characterization defined fibroblast heterogeneity at the point of enzymatic digestion (passage 0; P0) in both tumour-derived and non-cancerous lung (NCL) fibroblasts, establishing subset distributions within non-small cell lung cancer (NSCLC). Notably, CAF-S1, CAF-S4, and CAF-S5 were enriched in tumour-derived fibroblasts, while CAF-S3 phenotype, although present in tumours, was more abundant in adjacent non-cancerous lung, where it was the predominant subset. CAF-S2, however, was found across both tissue types.

**Fig. 2:**
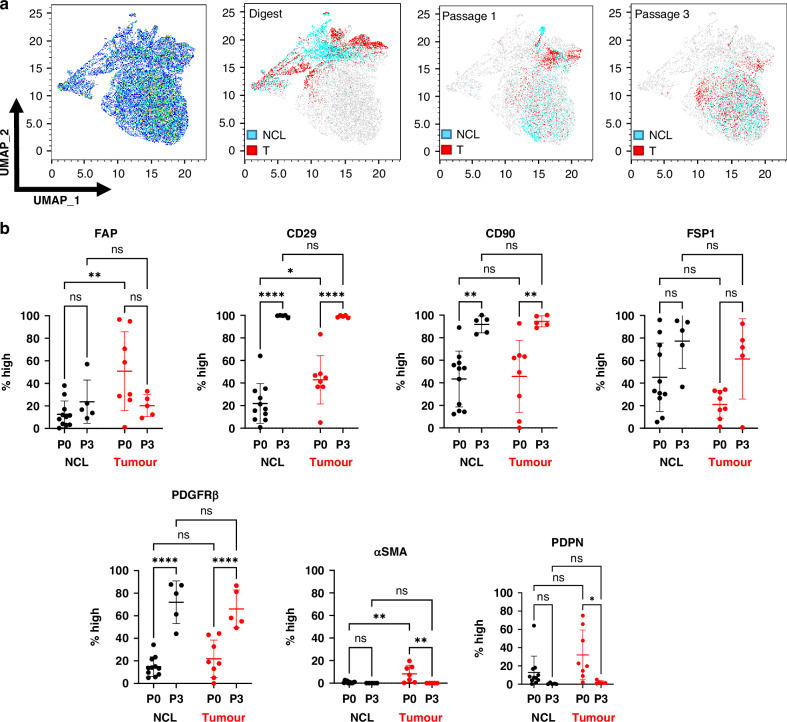
Expression levels of fibroblast activation markers change in culture. **a** UMAP demonstrating the heterogeneity of cells following digest, with distinct regions identified as tumour (T) and non-cancerous lung (NCL) which become less distinct by passage 1 and are mixed at passage 3; **b** Expression levels, expressed as proportion of fibroblasts demonstrating high levels of each marker comparing expression at passage 0 (following tissue digest) and passage 3 for both non-cancerous lung (NCL) and tumour. *N* = 11 P0 NCL, *N* = 8 P0 T, *N* = 5 P3 NCL & T. Error bars show mean with standard deviation. Statistics show ANOVA with Tukey’s multiple comparisons test, ns=not significant, *p < 0.05, **p < 0.01, ***p < 0.005, ****p < 0.001.

To investigate the phenotypic stability of these fibroblast populations in culture, we systematically assessed marker expression at each passage. A uniform manifold approximation and projection (UMAP) analysis of all expression data (Fig. 2a) revealed distinct segregation between fibroblasts from tumour and NCL at P0. By passage 1, these populations exhibited increasing overlap, and by passage 3, fibroblasts from both origins largely clustered together, suggesting a convergence in marker expression rather than the selective expansion of a specific fibroblast subset.

Next, we quantified the proportion of fibroblasts exhibiting high expression of each marker [6] from tumour and NCL from digest (passage 0) up to passage 6. This demonstrated differences at P0 between populations (Fig. 2b), but by passage 3, the expression levels had converged (Fig. 2b) and remained stable over subsequent passages (Fig. S2). Notably, FAP, a hallmark of fibroblast activation, was initially upregulated in tumour-derived fibroblasts compared to NCL fibroblasts, but by passage 3, no significant difference persisted. Instead, we observed an upregulation of FAP expression from baseline in NCL fibroblasts, coupled with a reduction in tumour-derived fibroblasts, reinforcing the shift toward a shared phenotype. Additionally, CD29, CD90, and PDGFRβ exhibited significant upregulation in culture across all fibroblast populations, irrespective of their tissue of origin. Conversely, αSMA and PDPN, which were expressed in fibroblasts at P0, were progressively lost in culture, supporting that standard culture conditions drive a homogenized fibroblast phenotype.

**Fig. 3:**
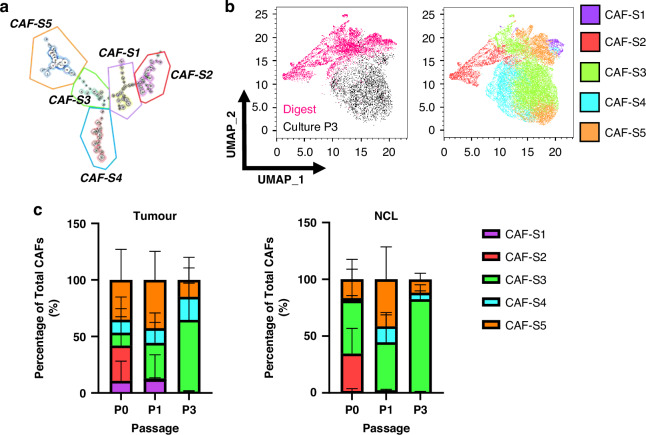
Changes in CAF subset heterogeneity throughout culture. **a** FlowSOM schematic of fibroblasts from tumour and NCL at digest (pink) and passage 3 culture (black) compared to UMAP locations of CAF subsets previously defined in Mathieson et al. for NSCLC; **b** Location of CAF subsets on a UMAP of the data in this study; **c** Proportions of each CAF subset passages 0, 1 and 3 for both tumour and non-cancerous lung (NCL). *N* = 10 P0, *N* = 4 P1, *N* = 5 P3. Error bars show standard deviation.

Several studies highlight the significance of identifying CAF subsets; therefore, we evaluated the changes in culture using the five subsets previously identified (Fig. 3a) the UMAP analysis demonstrated the fibroblasts from digest demonstrate representation from all five (S1–S5) subsets (Fig. 3b). In contrast, the predominant population in culture, particularly by passage 3, is the S3 subset (FAP^Low^ CD29^Med^ αSMA^Neg-Low^ PDPN^Low^ CD90^Low^ FSP1^High^ PDGFRβ^Low^), irrespective of tissue origin, with partial overlap with S4 and S5.To assess this quantitatively, we plotted the proportion of total fibroblasts for each tissue type that was represented by each subset for initial expression (P0), passage 1 and passage 3 (Fig. 3c). In CAFs, this demonstrated heterogeneity across all five subsets initially, but by passage 1 there is a loss of CAF-S2 and by passage 3, CAF-S1 is lost and CAF-S3 dominates. In NFs, we initially observe no CAF-S1 or CAF-S4 in the population, with CAF-S2 and CAF-S3 dominating; however, we also observe a loss of CAF-S2 at passage 1 and dominance of CAF-S3 by passage 3.

**Fig. 4:**
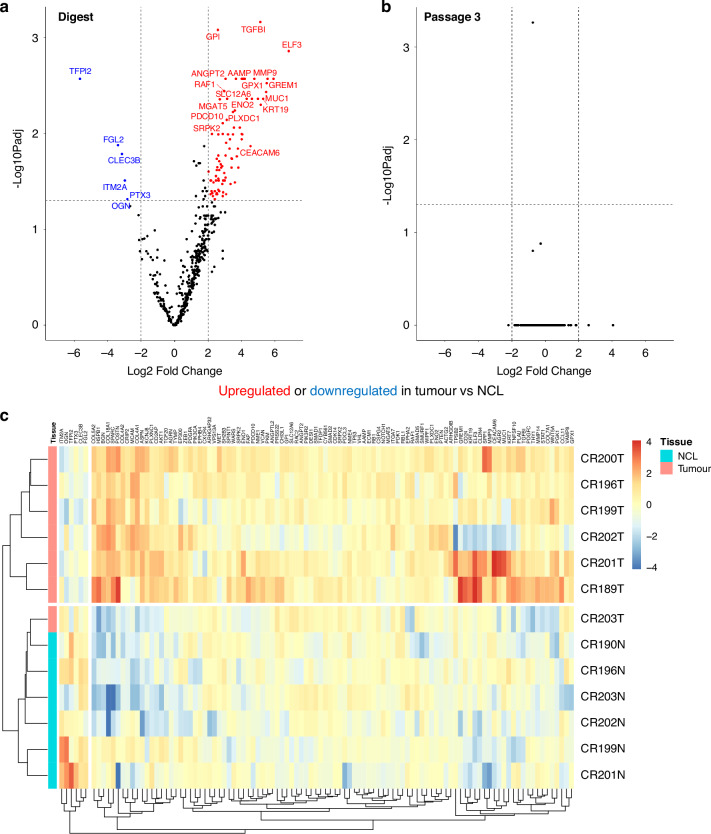
Transcriptional differences between fibroblasts isolated from tumour and non-cancerous lung. **a** Differences between tumour and NCL post-digest; **b** Differences between tumour and NCL after passage 3; **c** Heat map showing the 100 most differentially expressed genes at digest.

To understand the changes on a transcriptional level, bulk RNA sequencing using the NanoString Human PanCancer Progression panel was performed on flow sorted fibroblasts following initial tissue digest (passage 0), and fibroblasts which had been maintained in culture until passage 3. Fibroblasts isolated from tumour and NCL samples display transcriptional differences ex vivo, demonstrated in Fig. 4a, c, where several genes such as TGF-β1, a key regulator of fibroblast activation, and MMP9 are upregulated in the CAFs compared to NCL fibroblasts (comparison within tissue types shown in Supplementary Fig. S3). However, notably at passage 3 we see no significant transcriptional differences were observed between fibroblasts of different origins, suggesting that they have attained a convergent transcriptional profile. Pathway enrichment analysis (KEGG and GO) revealed activation of PI3K-Akt, ECM-receptor, and HIF-1 signalling pathways, with low-level enrichment of autophagy-related terms, suggesting engagement of stress adaptation mechanisms during in vitro culture (Fig. S4)

**Fig. 5:**
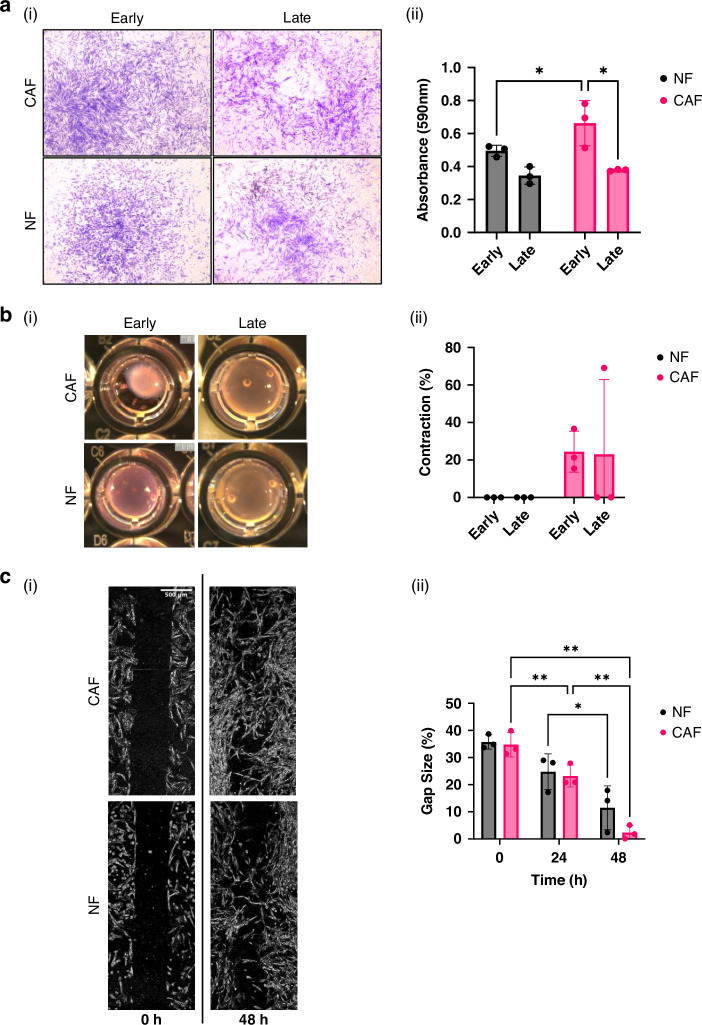
Functional assessment of fibroblasts derived from tumour and non-cancerous lung at different stages of culture. **a** Transwell migration assay (i) Representative images showing fibroblasts that have migrated across the transwell membrane stained with crystal violet for quantification; (ii) Comparison of migratory ability between early and late passage for CAFs and NFs (higher absorbance reading indicates more cells migrated); **b** Collagen contraction assay (i) Representative images showing comparison of contractile ability of early CAFs and NFs; (ii) Comparison of contractile ability between early and late passage of CAFs and NFs; **c** Gap closure migration assay (i) Representative images showing the initial gap between fibroblasts (left at 0 h) and after 48 h when the fibroblasts have migrated to close the gap (scale bar 500 μm); (ii) Gap size proportions after 0, 24 and 48 h for CAFs and NFs at late passage. *N* = 3 independent CAF lines for all assays, statistics shown are uncorrected Fishers LSD for A and B, and Tukey’s multiple comparisons test in C, *p < 0.05, **p < 0.01.

To determine if there were functional changes in fibroblasts at different stages of culture, early (P1-2) and late passage (*P* ≥ 3) fibroblasts from tumour (CAFs) and NCL (NFs) were compared in migration and contraction assays. Transwell migration assays (Fig. 5a) revealed that in both CAFs and NFs, fibroblasts possess greater migratory ability at early passage. When comparing differences between CAF and NF at each stage, there was greater migration in early passage CAFs compared to early passage NFs. When investigating contractile ability using the collagen contraction assay (Fig. 5b), it became evident that NFs possess no contractile ability at any stage of culture (Fig. 5bii). CAFs potentially retain some ability to be contractile in culture, but this was only demonstrated in one sample at late passage. We also monitored fibroblast migration over time using a gap closure assay (Fig. 5c) and recording the gap size (as a proportion of the whole image) every 24 h (Fig. 5cii). This did not demonstrate any significant differences in migratory ability between CAFs and NFs at any time point but a reduction in the gap size over time. Finally, we looked at the secretome of cultured fibroblasts, comparing between passage 3 CAFs and NFs across 14 factors (Supplementary Fig. S5). No significant differences were found between cell types across a range of cytokines and chemokines including TGF-β, IL-6, CXCL12 and CCL2.

## Discussion

We have demonstrated that cancer associated fibroblasts from NSCLC undergo phenotypic drift in culture. We have shown that irrespective of lung tissue origin (tumour or non-cancerous lung), fibroblasts in vitro show convergence of key CAF markers. Using previously defined NSCLC CAF subset classifications for CAF-S1–S5 [6], we have shown that conventional in vitro conditions, the predominant CAF phenotype that emerges is the CAF-S3 subset as early as passage 3, regardless of the initial distribution of CAF subsets identified in the samples. This is associated with convergence of the transcriptome and loss of functionality after culture, demonstrated by a loss of migratory ability.

Three markers were found to be upregulated in culture conditions, regardless of tissue origin: CD29, CD90 and PDGFRβ. CD29 (integrin β1) is expressed on numerous cell types, and has roles in cellular adhesion, interacting with the ECM and cell-cell adhesion [36, 37]. This upregulation may be due to being grown adherent to plastic cell culture flasks, where adhesion molecules would be upregulated. This has been observed in T84 human colorectal cancer cells, where growing on a rigid plastic surface upregulated CD29 compared to when cells were cultured on a soft collagen gel substrate [38]. CD90 is also an adhesion molecule expressed on numerous cell types, including fibroblasts, and has been used as a marker on cultured fibroblasts to eliminate them from culture of other primary cells [39]. Similar to CD29, the increase in CD90 expression could be due to fibroblasts being grown on adherent surfaces. PDGFRβ contributes to fibroblast roles in migration, regeneration and wound healing, modulating processes such as ECM production and angiogenesis [40]. Given that in response to wound signalling, fibroblasts enter an activated state allowing for increased ECM production to heal the wound, this upregulation of PDGFRβ in culture suggests that the fibroblasts are maintained in an activated phenotype. This finding is further supported by the expression of FAP in culture shown here. In fibroblasts from NCL tissues, FAP is expressed at low levels, whereas in CAFs from tumour tissue high levels of FAP expression can be identified (this has been shown and discussed in our previous study [6]). However, in culture, we observe moderate expression of FAP in fibroblasts from both tissue types, suggesting that in culture, the fibroblasts are maintained in an activated state, although potentially not as strongly activated as those found in the NSCLC tumour microenvironment.

Both αSMA and PDPN show a complete loss of expression on fibroblasts by passage three in culture, regardless of tissue origin. A previous study investigating CAFs from invasive ductal carcinoma of the pancreas investigated the impact of different culture conditions on levels of PDPN expression [41]. They found that PDPN expression decreased in culture when high levels of serum were used. In our study, culture medium contained 10% FBS, which could have influenced the loss of PDPN expression. Therefore, serum free culture conditions may be required to maintain PDPN expression levels. CAFs that positive for αSMA in the native tumour microenvironment have been shown to be located closer to the tumour cells than those not expressing it in NSCLC [6], PDAC [42] and breast cancer [22]. Therefore, as the populations in culture become pure fibroblast cultures and any epithelial cells are lost, cells may not be promoted to express αSMA. This loss of αSMA expression likely also contributes to the loss of migratory and contractility of the CAFs in late passage culture given that its expression is associated with these functions [43].

The identified changes in marker expression levels collectively result in a S3 phenotype, irrespective of tissue origin, suggesting a mildly activated fibroblast state. Previous studies examining CAF phenotypes have employed functional comparisons of subsets, often involving flow sorting followed by specific culture conditions. For instance, Pelon et al. cultured CAFs derived from breast cancer to distinguish their CAF-S1 and CAF-S4 subsets, using pericyte medium and culturing under a humidified atmosphere of 1.5% O_2_ and 5% CO_2_ [44]. Similarly, Givel et al. successfully cultured the CAF-S1 subset from ovarian cancer by allowing fibroblasts to grow out from tumour tissue pieces rather than from single-cell suspensions [24]. Croizer et al. further refined CAF subset cultures from breast cancer using collagen-coated surfaces to enhance fibroblast adherence and growth conditions [45]. These findings suggest that with customised culture adaptations, it may be feasible to culture CAF subsets.

We have demonstrated that TGF-β1 is upregulated in CAFs, serving as a central mediator of fibroblast activation and phenotypic remodelling. Through both canonical SMAD-dependent and non-canonical pathways, TGF-β1 regulates key processes such as myofibroblast differentiation, extracellular matrix (ECM) production, and cellular plasticity [2, 3]. It also promotes autophagy [46], which may drive tumour-derived CAFs toward a mildly activated fibroblast state, contributing to the transcriptional and functional homogenization observed between fibroblast populations.

Our transcriptomic data show enrichment of PI3K-Akt, HIF-1, and ECM-receptor interaction pathways, which are regulators of fibroblast plasticity and survival [47]. Additionally, we observed modest enrichment of autophagy-related pathways which may represent adaptive mechanisms to in vitro culture conditions, and contribute to the phenotypic convergence observed in both CAF and NF populations. The enrichment of autophagy-related pathways is particularly notable, given the known interplay between fibroblast activation and metabolic stress. Previous studies have shown that CAFs exposed to nutrient deprivation or stiff 2D substrates engage autophagic programs to support survival and maintain secretory function [48, 49]. In our model, both CAFs and NFs are removed from their native microenvironments and subjected to culture conditions, making it plausible that autophagy contributes to their phenotypic convergence. With optimized culture adaptations, it may be feasible to maintain or even expand CAF subset identities in vitro.

While our study focused on characterizing phenotypic and functional changes during fibroblast culture, we did not directly investigate the molecular mechanisms driving this plasticity. Nor did we test strategies to maintain primary fibroblast phenotypes, such as alternative media, substrate coatings, or advanced systems like 3D cultures. The potential to reverse culture-induced phenotypic drift or to rescue specific fibroblast states also remains unexplored. Nonetheless, the upregulation of TGF-β1 and enrichment of autophagy-related pathways point to conserved stress-response mechanisms. Both TGF-β signalling and autophagy are established mediators of fibroblast adaptation to metabolic and mechanical stress and may underlie the convergence observed in vitro. Future studies incorporating mechanistic analyses will be essential to identify regulators of CAF subset stability and inform approaches to preserve heterogeneity in culture.

Our findings contribute to the growing understanding that fibroblast phenotypes are highly plastic and context-dependent. In vivo, CAF states can shift dynamically in response to tumour-derived cues and mechanical stimuli, as demonstrated by recent studies employing organoids, co-culture systems, and spatial transcriptomics [2, 50]. These dynamic transitions help explain the heterogeneity observed among CAFs in the tumour microenvironment and highlight the limitations of conventional static culture systems. Future strategies for CAF modelling may benefit from incorporating 3D matrices, hypoxia, or tumour-conditioned media to better replicate native microenvironmental conditions.

In summary, we have demonstrated that cancer-associated fibroblasts (CAFs) and fibroblasts from non-cancerous lung (NCL) tissue adopt a similar phenotype when cultured under standard in vitro conditions. Our findings therefore, highlight the importance of characterising any phenotypic changes that occur during culture, particularly when comparing CAFs to fibroblasts from non-cancerous lung tissue. This is significant because the convergence of phenotypes observed in culture does not reflect the true diversity of fibroblast subsets present within the native tumour microenvironment.

## Supplementary information

Supplementary Data

## Data Availability

Data from this study is available from the corresponding author upon request. Nanostring nCounter data is available 10.7488/ds/7767. All other data is available from the Authors.
