# Supplementary material for: Patient derived cancer-associated fibroblasts from non-small cell lung cancer undergo phenotypic drift in culture
Source: BJC Rep. 2025 Jul 10;3:50. doi: 10.1038/s44276-025-00159-w (PMC12246254; doi:10.1038/s44276-025-00159-w)
Supplement: Supplementary file 1 — Supplementary Data [file 44276_2025_159_MOESM1_ESM.docx]

**Supplementary Data**

Patient derived cancer-associated fibroblasts from non-small cell lung cancer undergo phenotypic drift in culture

Layla Mathieson^1,^, Phoebe Jones^1^, Lilian Koppensteiner^1,^, Liam Neilson^1^, David A Dorward^2^, Richard O’Connor^1,^, Ahsan R Akram^1,3^

^1^Centre for Inflammation Research, Institute of Regeneration and Repair, University of Edinburgh, 5 Little France Dr, Edinburgh BioQuarter, Edinburgh, United Kingdom EH16 4UU.

^2^Department of Pathology, Royal Infirmary of Edinburgh, Edinburgh, United Kingdom.

^3^Cancer Research UK Scotland Centre, Institute of Genetics & Cancer, The University of Edinburgh, Crewe Road South, Edinburgh, United Kingdom, EH4 2XR.

**Table S1: Antibodies used for flow cytometry staining.**

| Marker | Colour | Supplier | ul/test | Catalogue No. | Isotype | ul/test | Iso Catalogue No. |
| --- | --- | --- | --- | --- | --- | --- | --- |
| CD45 | BV605 | BioLegend | 5 | 368524 | IgG1 M | 5 | 400161 |
| CD31 | BV605 | BioLegend | 5 | 303122 | IgG1 M | 5 | 400161 |
| EpCAM | BV605 | BioLegend | 5 | 324224 | IgG2a M | 5 | 400349 |
| CD90 | VioBlue | Miltenyi | 2 | 130-119-890 | IgG1 M | 2 | 130-113-767 |
| Zombie | UV | BioLegend | 1 | 423108 | NA | NA | NA |
| FAP | APC | R&D | 5 | FAB3715A | IgG1 M | 5 | IC002A |
| PDGFRβ | AF594 | R&D | 5 | FAB1263T | IgG1 M | 5 | IC002T |
| CD29 | AF488 | BioLegend | 5 | 303016 | IgG1 M | 5 | 400129 |
| PDPN | APC-Cy7 | BioLegend | 5 | 337030 | IgG2a R | 2.5 | 400524 |
| αSMA | AF750 | R&D | 5 | IC1420S | IgG2a M | 5 | IC 003S |
| FSP-1 | PE | BioLegend | 5 | 370004 | IgG1 M | 5 | 400139 |

**
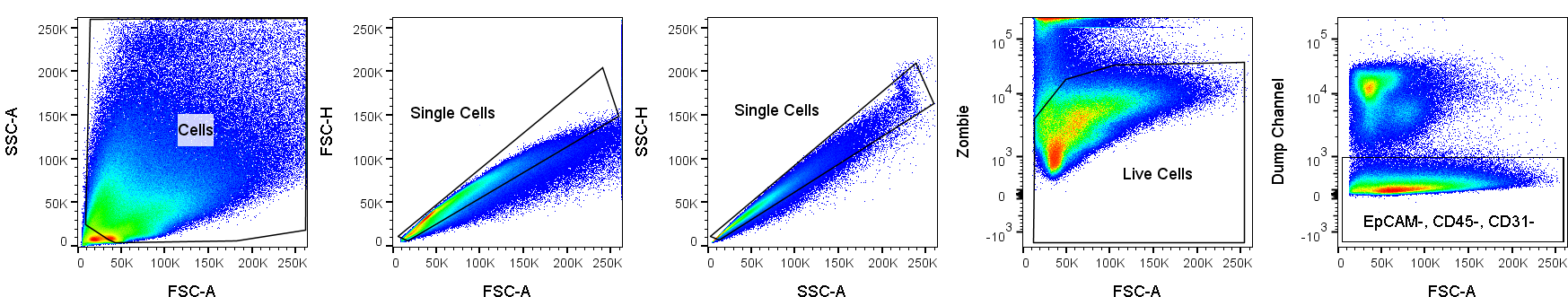
**

**Figure S1: Gating strategy used to define fibroblasts as single, live cells, which are EpCAM-, CD45-, CD31-.**

**Figure S2: Changes in fibroblast activation marker expression levels throughout passage measured from initial sample digestion (P0) to culture passage 6.** N=11 NCL P0, N=5 NCL P1-P3, N=4 NCL P4-P6, N=9 Tumour P0, N=4 Tumour P1, N=5 Tumour P2-P6. Error bars show standard deviation.

**
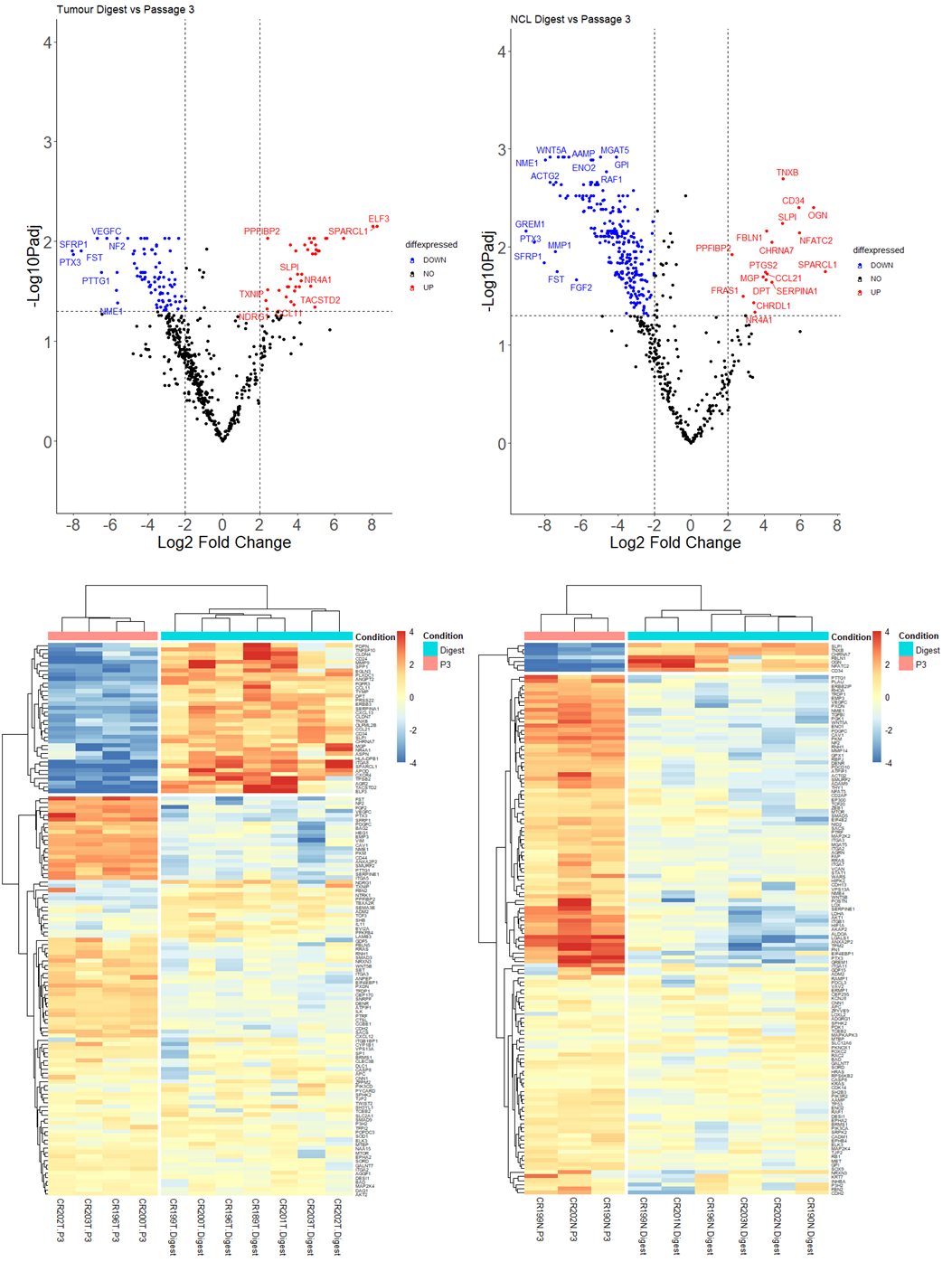
**

**Figure S3: Transcriptional differences between fibroblasts at digest and passage 3 shown for both tumour and NCL.** Volcano plots show differential expression of genes for tumour and NCL, comparing digest fibroblasts to passage 3. Genes which are significantly downregulated are shown in blue and up in red. Heat maps show the top 100 differentially expressed genes for each tissue origin, demonstrating clustering of digest fibroblasts and cultured fibroblasts in both tumour (left) and non-cancerous lung (right).


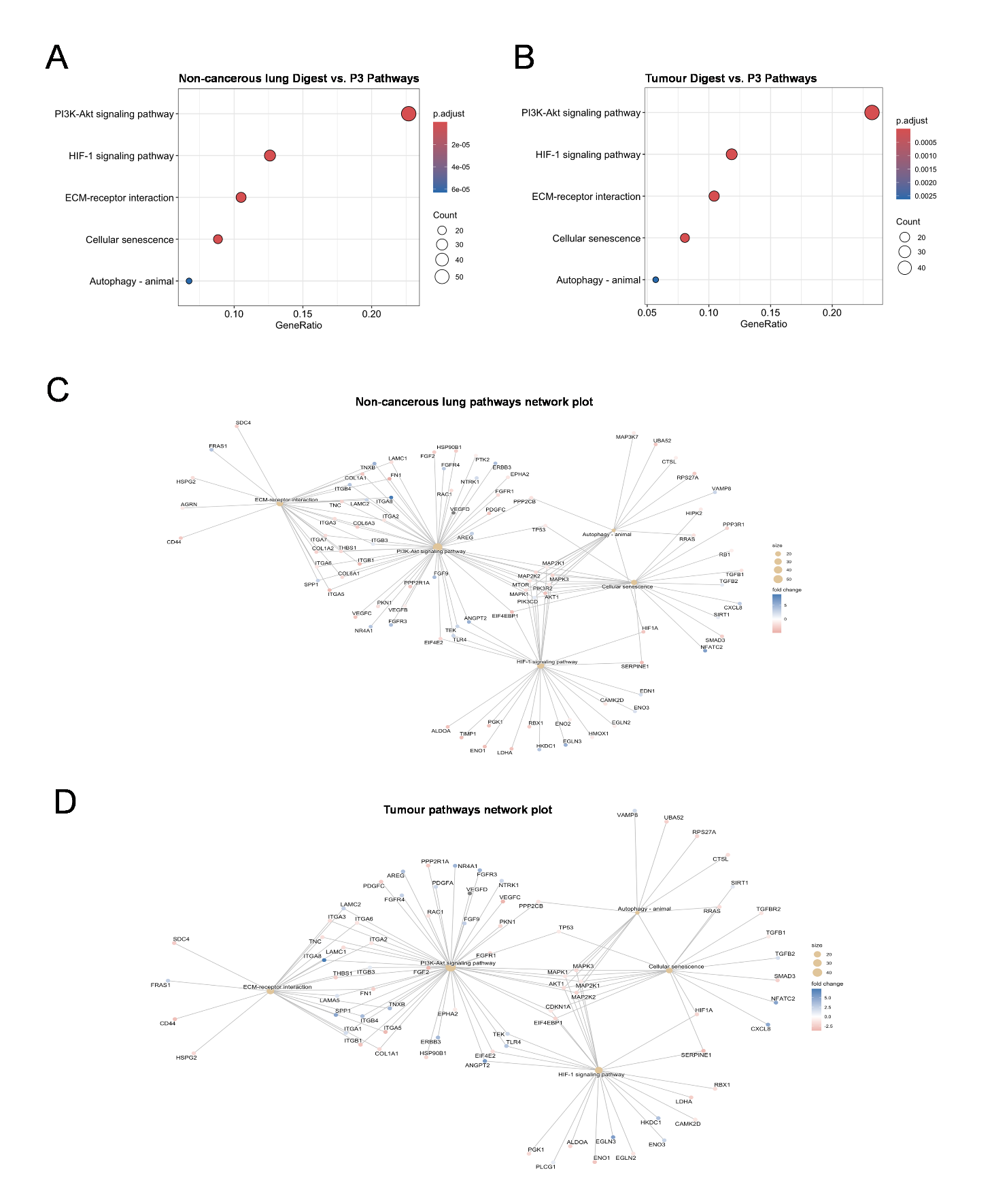
**Figure S4: KEGG analysis of Stress and Autophagy Pathways.** Dot plots display KEGG pathway enrichment in differentially expressed genes between freshly isolated (Digest) and passage (P3) fibroblasts from matched non-cancerous lung (A) and tumour samples (B). Network plots for non-cancerous lung (C) and tumour (D) demonstrate stress and autophagy pathways. Large nodes in yellow represent pathways, size of node indicates number of genes in pathway differentially expressed. Smaller nodes represent genes in these pathways and colour of smaller nodes indicates fold change in expression of genes.

**Figure S5: Comparing the secretome of passage 3 CAFs and NFs. All factors were measured using a custom Biolegend LegendPlex kit except LIF which was measured by ELISA.** N=4, error bars show standard deviation.
